# Sanitary Specialist Tadayasu Sakamoto (★ 1937 † 2021)

**DOI:** 10.1590/0037-8682-0620-2022

**Published:** 2023-02-20

**Authors:** Pedro Luiz Tauil

**Affiliations:** 1 Universidade de Brasília, Núcleo de Medicina Tropical, Brasília DF, Brasil.

The pharmacist-sanitary doctor Tadayasu Sakamoto was born in Jataizinho, Paraná, on October 13, 1937, and died in Brasília, DF, on December 28, 2021.

**Figure:**
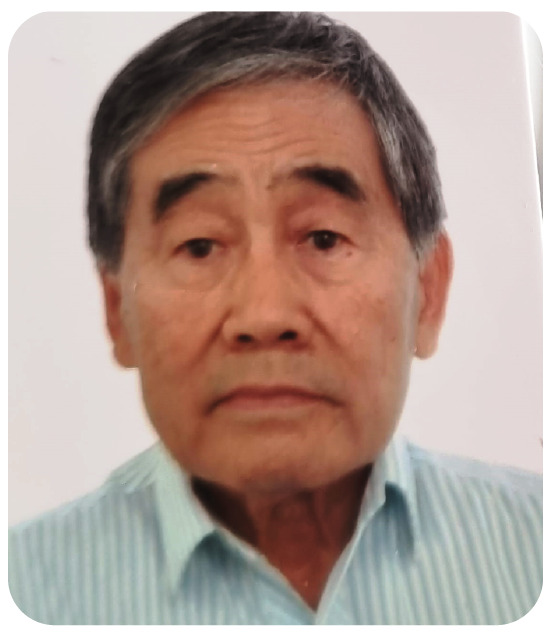

Sakamoto retired from the Superintendence of Public Health Campaigns of the Ministry of Health (SUCAM) on May 9, 1992. He graduated from the Faculty of Pharmacy of the Federal University of Paraná in 1962 and then took a specialization course in Food Control and Drugs at the same faculty in 1963. He took other specialization courses, including Malariology, at the Faculty of Hygiene and Public Health, University of São Paulo, in 1965, and the Public Health course at the National School of Public Health at Oswaldo Cruz Foundation in Brasilia, DF, in 1978.

Sakamoto began his professional career in controlling endemic diseases in Santa Catarina, where he participated in the control of malaria, drastically reducing its incidence in the state. He became the Regional Director of SUCAM in 1977 and in 1979 he was transferred to the headquarters of SUCAM in Brasília and designated the head of Field Operations of the Chagas Disease Division of the Department of Endemic Eradication and Control (DECEN). It was at that moment that I started working with him professionally, as I was the Director of DECEN. I was then able to assess their work in the control of Chagas Disease carried out together with the Head of the Division, Dr. Antonio Carlos Silveira. 

At that time, the National Program for the Control of Chagas Disease was expanded for the first time to the entire area known to be endemic for the disease in Brazil. This growth was very successful, drastically minimizing the transmission of the disease by reducing its main vector in Brazil, *Triatoma infestans*. This success continues on to this day, as this vector has practically been eliminated. Antônio Carlos Silveira and Tadayasu Sakamoto led this entire process, selecting and training personnel for the program, replacing the BHC insecticide with a pyrethroid, and keeping disease surveillance under control throughout the country. We owe the success of this program to these two sanitarians, who began to be exported to other countries, including Argentina, Uruguay, Chile, and Paraguay.

Sakamoto was a consultant for the Pan American Health Organization in Argentina in 1980 and Uruguay in 1983. He has published several works in the *Brazilian Journal of Malariology and Tropical Diseases* and in the *Journal of the Brazilian Society of Tropical Medicine*. Highlights of his work include, “On the focus of Triatomines domiciled in the Baixada Fluminense” (1982)^1^; “Medical-social importance of Chagas disease in Brazil and its control” (1983)^2^; and “Home application of BHC and concentration of the product in the blood of a population exposed to control in the Municipality of Alexânia, Goiás” (1984).

He also presented papers at conferences, such as “Experimental trial with Deltamethrin in the control of a household population of triatomines - preliminary data” at the XIX Congress of the Brazilian Society of Tropical Medicine (1983) and “Large Field Trial of Alternative Insecticides and Formulations for the Control of Chagas Disease Vectors - Preliminary Results” at the XIX Annual Meeting of Basic Research in Chagas Disease, Caxambu, MG (1985).

In addition to being colleagues, Tadayasu Sakamoto was a great friend. I am a witness to his competence as a sanitarian and his attention and kindness toward patients and co-workers in the control of endemic diseases in Brazil. 
